# DNA damage interactions on both nanometer and micrometer scale determine overall cellular damage

**DOI:** 10.1038/s41598-018-34323-9

**Published:** 2018-10-30

**Authors:** Thomas Friedrich, Katarina Ilicic, Christoph Greubel, Stefanie Girst, Judith Reindl, Matthias Sammer, Benjamin Schwarz, Christian Siebenwirth, Dietrich W. M. Walsh, Thomas E. Schmid, Michael Scholz, Günther Dollinger

**Affiliations:** 1 GSI Helmholtz Center for Heavy Ion Research, Department of Biophysics, Darmstadt, Germany; 2 TU München, Klinikum Rechts der Isar, München, Germany; 3 Institute of Innovative Radiotherapy, Helmholtz Zentrum München, Neuherberg, Germany; 4 Universität der Bundeswehr, München, Germany

## Abstract

DNA double strand breaks (DSB) play a pivotal role for cellular damage, which is a hazard encountered in toxicology and radiation protection, but also exploited e.g. in eradicating tumors in radiation therapy. It is still debated whether and in how far clustering of such DNA lesions leads to an enhanced severity of induced damage. Here we investigate - using focused spots of ionizing radiation as damaging agent - the spatial extension of DNA lesion patterns causing cell inactivation. We find that clustering of DNA damage on both the nm and µm scale leads to enhanced inactivation compared to more homogeneous lesion distributions. A biophysical model interprets these observations in terms of enhanced DSB production and DSB interaction, respectively. We decompose the overall effects quantitatively into contributions from these lesion formation processes, concluding that both processes coexist and need to be considered for determining the resulting damage on the cellular level.

## Introduction

Among DNA lesions, double strand breaks (DSB) formed by two single strand breaks (SSB) in close proximity on the scale of some nanometers are the key elementary lesions for cell inactivation^1,2^ and induction of mutations^3,4^. DSB are frequently induced by reactive oxygen species or other oxidative stress, by interaction with chemicals^5^ or radiation^6^, by replication stress or are even stimulated by cellular processes within certain phases and types of eukaryotic cell division like meiosis^7^. DSB stimulated by these processes are usually repaired with high fidelity by various repair pathways. This is also valid for DSB induced by ionizing radiation^2^, but there a relatively larger fraction remains unrepaired or gets misrepaired leading to e.g. chromosomal aberrations or gene mutations^4^. This suggested the relevance of complex damage, where in general DSB interaction with further, proximate damage is meant to be a substantial contribution for cell inactivation. The interaction partner could be in some basepairs distance, but also possible DSB-DSB interaction within a µm range is discussed^8^.

Considering radiation as a damaging agent, it is well known and supported by a plethora of dedicated experiments that the radiation effect to cells and tissues is not uniquely determined by the average energy dose to which the cells are exposed. For instance, in comparison with photon radiation, heavy ions are more effective in stopping cell proliferation and inducing mutations^3,9,10^ which is usually attributed to the highly localized energy deposition along their path: There, secondary electrons are produced at a high rate, carry the energy outwards and give rise to the formation of a corridor of high ionization density - the so-called track structure - whose lateral dose profile provides very high local doses of up to about 10^7^ Gy. Such high ionization densities cause a larger number of induced DSB per Gy organized along tracks^11^, cause ‘dirty’ DNA ends at the DSB, and facilitate the interaction of neighbored DSB as they are induced spatially correlated. Therefore the energy loss of radiation per path length, also expressed as linear energy transfer (LET), is an important factor parameterizing the resulting biologic effect. In contrast to such high-LET radiation, for low LET ion radiation, as e.g. for high energetic protons, local doses are moderate. Thus DNA lesions are distributed much more homogeneous, similar to DNA lesions induced by X-ray irradiation covering more homogeneously the exposed volumes. It is thus commonly accepted that the lesions induced by high LET irradiation are much harder to repair than clean ends or individual, well separated DSB, resulting in a larger number of e.g. chromosomal aberrations^6^.

From a formal perspective, a coexistence of any two elementary lesions in sufficient proximity can act synergistically and result in a more complex lesion whose effect is larger than the sum of the effects of both isolated lesions. Such interaction mechanisms go along with a spatial scale of proximity and an associated biologic target, i.e. a conformation level of the DNA^12^ or a characteristic scale for mobility for the induced damage in random walk processes. So far there is no general answer on how the overall observed effect modifications are affected by processes on different levels of damage localization.

In the literature the relevance of the nanometer scale has been investigated, stimulated by the formation processes of DSB. This has led to the notion of complex DSB, where the complexity refers to additional lesions within some nm distance^1,13,14^. In contrast, also the µm scale is known to have relevance since studies on the interaction range for chromosome aberrations^15^, and is also given different interpretation in terms of DNA conformation^16^. Also other scales of lesion interaction and the possible coexistence of scales were proposed^17^. Independent of this a number of studies investigated the phenomenon of sublethal damage, where lethality was committed only after a second dose of the damaging agent^18,19^. However, a rigorous investigation of which scales are of importance and what their relative contribution is in different experimental settings is lacking. This work presents an experimental and modelling approach to disentangle the relevance of the different scales of DNA damage.

## Results

### Experiments

In our experiments, we analyzed the impact of DNA damage induction on CHO cells using cellular survival level measured by colony formation as endpoint, which is regarded as a ‘gold standard’ in radiation biology. Clonogenic cell survival is a clean and representative endpoint which reflects the final level of damage after all cellular repair or misrepair processes have been completed.

As DNA damaging agent we have used ionizing radiation, allowing to vary the spatial distribution of damages by the appropriate choice of radiation type and irradiation conditions. As reference case we have chosen conditions where DNA damages are distributed throughout the target in an approximately random manner, thus maximizing the mean distance between individual DSB and minimizing the probability of mutual interaction. This condition is fulfilled for low-LET radiation like e.g. photon radiation. Identical damage distribution patterns can be achieved also with high energetic protons using conventional broad beam irradiation (i.e. random distribution of particle traversals), if the mean number of DSB induced per proton traversal through the cell is significantly below 1.0. According to the minimized DSB interaction probability under these conditions, allowing for optimal repair of radiation damage, photon and high energy proton radiation are expected to exhibit the lowest effectiveness.

We have compared these reference conditions with situations where DSB were induced in a clustered, spatially correlated manner, using the following options:

- The concentration of particle traversals in small, subcellular regions, leaving the space in between these irradiation spots unaffected; in this case interaction can occur between DSB induced by different ion tracks (inter-track interaction) within the spots, while the total number of DSB is kept constant.
- An increase of the energy deposition per particle traversal and with that the number of DSB induced per traversal e.g. by choosing heavier particles like Li or C ions. In this case, damage interaction can occur between DSBs of a single track, which are produced preferentially along the trajectory of the ion.
- A combination of the two above mentioned options.

In our experiments we have realized all these options by using the capabilities of the ion beam microprobe SNAKE^20,21^, where a defined number of ion traversals can be applied either in a homogenous, random distribution or in sub-micrometer sized focused beam spots^22^. For the latter, electrostatic deflection of beam paths is employed to direct individual particles to pre-specified locations (see details in Methods).

**Figure 1:**
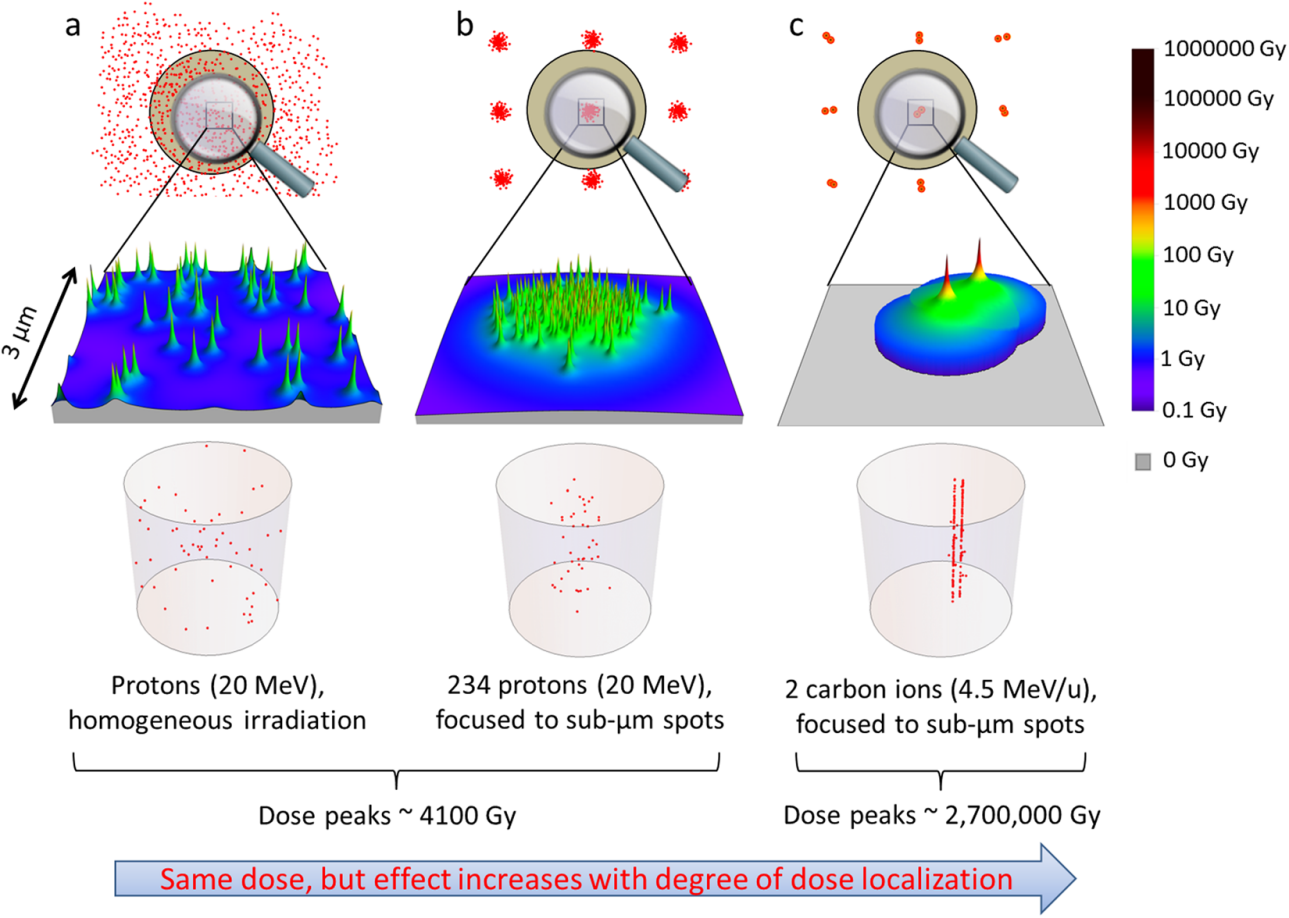
Experimental approach of investigating DNA lesion interaction with bunched radiation spots. The concept of changing dose distributions (upper) and DSB distributions (lower) on cell nuclei using different application patterns and different kind of ions while keeping the same mean dose is schematically visualized in a logarithmic color-scale and as schematic 3D plots, respectively. In comparison to a random beam application of low LET protons (**a**), microbeam bunching of the particles to µm size spots promotes interaction of DSB induced by different tracks (**b**), and bunching of high LET carbon ions at equal dose provides an extremely high energy concentration on the nm scale in the interior of their tracks (**c**).

In a first step, we have compared the effects of randomly distributed low LET proton traversals (acceleration energy 20 MeV, LET = 2.66 keV/µm at the cell nucleus) with spatially bunched proton spots, where the mean dose was 1.7 Gy in both conditions. The corresponding local energy deposition patterns and DSB distributions are depicted schematically in Fig. 1a,b. The spots were formed by narrow Gaussian microbeams. The bunched proton spots were arranged in a regular grid to irradiate a large area, and the number of particles per spot was adapted to keep the mean dose constant.

In a second step, we have used bunched high LET particle irradiation (33 MeV Li ions, LET = 81 keV/µm; and 55 MeV carbon ions, LET = 338 keV/µm) with its pronounced local dose distribution on the nm scale (Fig. 1c), leading to the induction of spatially correlated damages along the ion trajectories, as was exploited in preliminary experiments for chromosome aberrations^23,24^. For protons this is not the case as the number of DSB induced per track is well below 1.0.

In the experiments the Gaussian beams were elliptical with approximately 0.6 µm and 1.2 µm FWHM; the same spot geometry was used for all experiments described. In order to vary the number of ions per spot and to correspondingly vary the local DNA damage density, different mesh widths were applied, where an increase in the mesh width was compensated by a corresponding increase in the number of ions per spot to keep a mean dose of approximately 1.7 Gy fixed. The different beam delivery modes were verified with a DNA damage marker (c.f. Fig. S4). For instance, 117 protons were focused to each spot of a 5.4 × 5.4 µm^2^ grid. While for the smallest mesh width used in this study (3.82 × 3.82 µm^2^) on average about 4.6 spots hit the cell nuclei based on the mean nuclear area of 67.6 µm^2^ (standard deviation 24.8 µm²), for the largest grid (10.8 × 10.8 µm^2^) only 0.58 spots are applied on average to a cell nucleus and 44% of cell nuclei remain unhit.

Table 1 gives an overview of all radiation qualities studied, grid mesh widths, the ion numbers per spot and the exact mean dose applied to the irradiated cells. Moreover, the LET within an entire spot (“spot LET”) is given as the LET of a single particle times the particle number within the spot. Also the measured survival rates are listed. The expected numbers of DSB per particle traversal and per spot, calculated within the model framework to be described below, are provided in the supplementary information in Table S1. An overview of the amount of experimental data that has been retrieved is given in Table 2: Within each beamtime several spot irradiations have been realized, for each of which multiple samples have been irradiated. While such multiple irradiated samples within each beamtime allowed statistical assessment of the results, only results from different beamtimes are considered as independent experiments due to possible correlations from the common cell preparation.

**Table 1:** Overview of physical properties of the various combinations of ion species and microbeam grid variants. Spot numbers and number of particles per spot have been chosen to keep the dose approximately constant to 1.7 Gy.

Ion species	Grid mesh (µm^2^)	Ions per spot	Av. # spots per nucleus	Spot LET (keV/µm)	Mean Dose (Gy)	Mean cell survival	Survival uncertainty
Protons 20 MeV	broadbeam	—	—	2.66 ± 0.13	1.70 ± 0.09	0.76	+0.18−0.15
	5.4 × 5.4	117	2.32 ± 0.08	311 ± 16	1.71 ± 0.09	0.38	+0.10−0.08
	7.64 × 7.64	234	1.16 ± 0.04	621 ± 31	1.71 ± 0.09	0.41	+0.11−0.09
	10.8 × 10.8	468	0.58 ± 0.02	1242 ± 62	1.71 ± 0.09	0.43	+0.11−0.09
Lithium 33 MeV Li	broadbeam	—	—	81 ± 8	1.70 ± 0.17	0.187	+0.031−0.027
	3.82 × 3.82	2	4.63 ± 0.16	161 ± 16	1.77 ± 0.18	0.105	+0.026−0.021
	5.4 × 5.4	4	2.32 ± 0.08	322 ± 32	1.77 ± 0.18	0.091	+0.017−0.014
	7.64 × 7.64	8	1.16 ± 0.04	645 ± 65	1.77 ± 0.18	0.114	+0.022−0.018
	10.8 × 10.8	16	0.58 ± 0.02	1290 ± 130	1.77 ± 0.18	0.274	+0.109−0.078
Carbon 55 MeV	broadbeam	—	—	338 ± 34	1.70 ± 0.17	0.178	+0.023−0.020
	5.4 × 5.4	1	2.32 ± 0.08	338 ± 34	1.86 ± 0.19	0.095	+0.013−0.011
	7.55 × 7.55	2	1.19 ± 0.04	676 ± 68	1.90 ± 0.19	0.167	+0.022−0.019
	10.65 × 10.65	4	0.60 ± 0.02	1353 ± 135	1.91 ± 0.19	0.39	+0.056−0.049

The LET values consider energy loss in material before the ions hit the cell nuclei, and their uncertainty was assumed as 5% for protons and 10% for Li and C ions. The uncertainty in the average spot number per nucleus arise from the standard error of the mean of the average cell nuclear area.

**Table 2:** Overview of experiments completed and experimental evaluation of spot sizes.

Beamtime	Ion species	# Samples						Δx (µm)	Δy (µm)	Method
		C	R	3.8 µm	5.4 µm	7.6 µm	10.8 µm			
Apr 2014	55 MeV C	3	—	—	4	4	5	0.68	1.19	Polycarbonate
Aug 2014	55 MeV C	4	—	—	5	4	5	0.73	0.79	Polycarbonate
Oct 2014	20 MeV p	5	5	—	5	5	5	0.61	1.11	FNTD
Nov 2014	20 MeV p	5	5	—	5	4	4	0.51	0.99	FNTD
Feb 2016	33 MeV Li	6	5	5	5	5	—	0.67	1.36	CR 39
Jul 2016	55 MeV C	6	5	—	4	—	—	—	—	—
Nov 2016	55 MeV C	6	6	—	5	—	—	—	—	—
Feb 2017	33 MeV Li	5	3	4	4	5	5	0.65	1.47	CR 39

The number of samples specifies the number of independent platings for sham irradiated controls (C), random irradiation (R) or spot radiation in mesh grids of different widths (approximate mesh width indicated in the table). The beam spots had elliptical shape with semi-axes Δx and Δy, and different methods were performed for spot size characterization.

**Figure 2:**
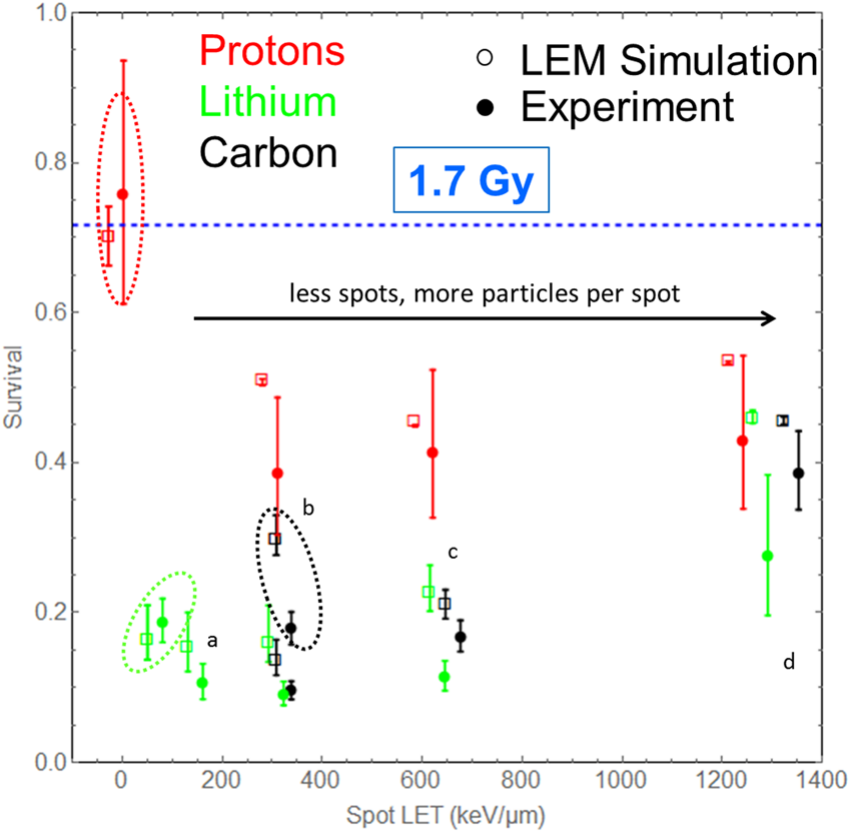
Cell survival vs the spot LET, i.e. the sum LET of all particles within a microbeam bunch for measurements (closed circles) and LEM simulations (open sqares) in comparison for different particles (p, Li and C in red, green and black, respectively) and different spot intensities. For all irradiations the dose was approximately 1.7 Gy (c.f. Table 1). Microbeam spots have been delivered as grids with mesh width of 3.82 µm (**a**), 5.4 µm (**b**), 7.64 µm (**c**) and 10.8 µm (**d**), where large mesh widths go along with larger particle numbers per spot. In addition, cell survival after broadbeam irradiation at 1.7 Gy is shown (data points are marked by dashed ellipses). The dashed blue line indicates the expected survival level after 1.7 Gy of X-rays. For better visibility simulation data points have been shifted by 30 keV/µm to the left. It is evident from the experiment that µm bunching enhances the effect, while at wider grids survival recovers again due to unhit cells. The simulations predict the survival in agreement with the measured data, supporting the underlying hypothesis. Note that the plot is shown in linear scale in order to present the differences at high survival most clearly.

Figure 2 displays the measured cell survival values plotted versus the spot LET. If two independent experiments from different beamtimes were performed (all cases except Li ions in a 10.8 µm mesh grid) the data points reflect average values (see Methods). Since a focused spot of low LET-radiation can be used to mimic properties of a single high-LET particle, we have chosen to plot the survival data on a linear scale against the spot LET to present the differences at high survival levels most clearly.

Impressively, simply by focusing protons to spots of 117 particles each (see Fig. 2, data points marked with ‘b’) dramatically reduces cell survival from 75% to about 40% as compared to the broadbeam application. This demonstrates that redistributions of energy deposition and consequently the damage patterns strongly influence the resulting effect. Unfortunately within the available beam time no data for protons in a smaller grid could be obtained. Also for lithium beams a small effect enhancing tendency is visible when comparing spot to random exposure. However, in this case already individual ions exhibit a much higher effectiveness, as indicated by the low survival observed after random broad beam irradiation; the same is true for carbon ion irradiation. These observations strongly support the idea of a synergistic interaction of DNA lesions on the µm scale, either between lesions induced by different ion traversals in the case of focused spot irradiation or between lesions induced within individual ion tracks in the case of heavier particles. It suggests that an enhanced effect observed for ions at least partially results from the geometric structure of the microscopic dose pattern and thus DNA damage distribution.

At the same spot LET, four lithium ions or a single carbon ion per spot can show an even higher effect, as cell survival is further decreased to only 10%. As the outer regions of the microscopic dose distribution within the tracks are comparable to that of an entire focused proton beam spot, this additional effect enhancement must emerge from the inner core of the tracks, where extremely high local doses are reached within some nm for Li and C ions but not for protons. Thus, the effectiveness of Li and C ions is also attributed to a synergistic combination of DNA lesions on the nm scale. As this combination is spatially restricted to the inner parts of the track, we use the term ‘interaction’ in analogy to the combination of lesions on the µm level.

Accumulating even more ions to the spots and exposing the nuclei to less spots to keep the dose constant only marginally modifies cell survival for protons, indicating a competition between increasing µm lesion interaction and the increasing number of unhit cells. The hit statistics even results in a restoration in cell survival for carbon and lithium beams when accumulating more particles per spot and widening the irradiation grid. For very large grids (marked with ‘d’ in Fig. 2) both proton and carbon irradiation effects saturate at about 40% cell survival probability, while the data point for Li ions stays somewhat below (note that here only measurements from one beam time are available). A saturation of survival is explained by recalling that cells which are hit at least once by one grid spot with a very high spot LET will be inactivated almost certainly, while unhit cells are expected to survive. Indeed a geometric consideration verifies that 44% of all cell nuclei remain without hits. The surprising observation that carbon ions irradiated in broadbeam are less effective than single carbon ions irradiated at fixed grid positions is also explained in that way, as in the former case large spaces and therefore unhit cells are more probable due to the randomness of the irradiation pattern. These findings directly demonstrate the importance of the 10 µm scale as defined by the nuclear size. In contrast to the nm and µm scale, the modification of the radiation action by means of the 10 µm scale occurs rather by the missing of lesions than by lesion interaction.

### Modelling

The experimental studies are accompanied with model calculations using the Local effect model (LEM)^25,26^, which have been performed as experiment independent ‘forward calculations’, i.e. in a predictive way, instead of as a fit model to only reproducing the measured data. Essentially, LEM predicts the effect of any concentrated lesion distribution pattern extrapolated from the rather homogeneous lesion distribution caused by high energetic photon radiation as a low LET reference case. It gives theoretic insight regarding the relevance of the different scales of DNA damage interaction and quantifies the importance of any of the relevant scales for the cell survival. It allows a prediction of cell survival after cell irradiation for any ion type, ion energy and applied hit pattern.

To quantify the radiation effect, three spatial scales of DNA damage formation are implemented in LEM and the corresponding processes are hypothesized to be relevant for the cellular survival after irradiation (for a visualization see Fig. S5): (i) On the nm level the interaction of SSB to DSB gives rise to an enhanced production of DSB per Gy for high local energy concentrations; (ii) on the µm scale the interaction of DSB within chromatin loops of the DNA 30 nm fiber leads to even more complex lesions which are harder to repair for the cell; (iii) on the 10 µm scale the hit statistics of a cell population may decrease the overall effect to correct for unhit cells. Notably, neither higher complexity levels of individual DSB on the nm scale nor the formation of chromosome aberrations are explicitly considered in the model, although the exploited effects of reference radiation imlicitly include the latter process. After all, LEM conceptually combines approaches developed in the framework of nanodosimetry^27^, microdosimetry^28^ and target theory^29^ on the three scales in a simple way, using only SSB and DSB as elementary DNA lesions. The model constants of a yield of 1250 SSB and 30 DSB per Gy and cell nucleus for photon radiation, a maximum distance of 25 bp for SSB to result in a DSB, and cubic domains according to 2 Mbp as size of chromatin loops for DSB interaction (leading to 510 nm side length considering the typical nuclear volumes) are taken as a fixed part of the model and were originally obtained by experimental data^30,25,31^. The lesion statistics predicted by this approach are shown in Table S1 along with further model details in the supplementary information.

The LEM has been adapted to the experimental conditions of this work by an implementation of the 2D Gaussian beam spots of a specified number of ions delivered to cells. The model results are displayed in Fig. 2 along with the experimental data. We found a qualitative and quantitative overall agreement with the measurements, although some differences persist. Notably, the simulations were performed completely autonomously without fitting to the measured focused ion beam effects: Only the photon dose response parameters (see supplementary information) and experimental characterization (ion type, energy, LET, spot beam dimensions, nucleus geometry) were used as model input.

### What determines the relative biological effectiveness?

Encouraged by the model agreement to the data we also analyzed systematically the relative impact of the considered mechanisms on the overall radiation damage with the model. The efficiency of radiation induced effects is usually expressed by the relative biological effectiveness (RBE), which is defined as the ratio of the dose *D*_*x-ray*_ needed for the induction of an effect by the high energy photon irradiation to the dose *D* where the same effect is obtained by the radiation type under investigation,

1 $$RBE=\frac{{D}_{x-ray}}{D}.$$

Considering that any of the three spatial scales pointed out potentially modifies the radiation effectiveness, the observed effects can be thought of being amplified by means of both SSB and DSB interaction and damped by hit statistics. This suggests an approach to disentangle the contribution of these mechanisms as

2 $$RBE=RB{E}_{{\rm{nm}}}\times RB{E}_{\mu {\rm{m}}}\times RB{E}_{10\mu {\rm{m}}}$$

where *RBE*_x_ is an effect modifying factor on the spatial scale x. In this approach *RBE*_nm_ is just the enhancement of the DSB yield, often referred as *RBE*_DSB_. The RBE_µm_ reflects the enhancement in the effect by the interaction of DSB on the µm scale. The factor *RBE*_10 µm_ is a hit statistic correction factor. It should be noted that the three *RBE*_x_ factors correspond to a hierarchy of processes. For example, a strong SSB interaction will result in more DSB and thus also amplify the DSB interaction. Hence RBE_µm_ depends implicitly on the value of RBE_nm_, and the processes are not independent, Nevertheless the factorization approach gives useful information as the terms are directly related to the individual processes. The different *RBE*_x_ therefore can be regarded as the relative contributions to the overall RBE, corresponding to number and proximity of DSB and to hit statistics. This implies that each of the underlying mechanisms has the capability to modify the RBE, and the factorization is a possible way of quantifying their relative importance.

**Figure 3:**
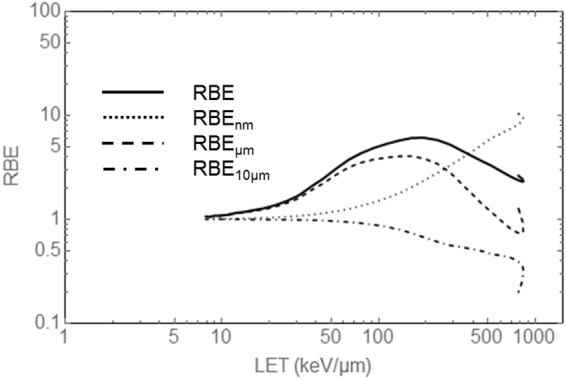
Contributions of the different interaction mechanisms to the total RBE_α_ for monoenergetic carbon ions according to Eq. 2 plotted versus LET. The curves have been calculated with the LEM for CHO cells as used in the described experiments. In the high LET region where particles come to rest and provide the highest energy deposition all three scales show a non-negligible contribution which has to be considered to understand the RBE.

The LEM allows an explicit evaluation of the *RBE*_x_, and Fig. 3 shows their contributions to the overall RBE of the cells under investigation versus the LET for carbon ions of different energy. We evaluated here the maximum RBE which occurs at low doses, often referred to as RBE_α_, and restrict to this limiting case, which reflects the modification of the initial slope of dose response curves. The overall RBE reveals the typical RBE-LET characteristics with an effect increase up to the pronounced maximum at 150 keV/µm. Beyond, the RBE decreases again due to the overkill effect. At the high LET end the hook occurs as for high LET values two values of kinetic energy are associated with the same LET, due to the distinct properties of ions decelerating in a surrounding medium^32^.

The DSB yield given by the *RBE*_nm_ factor increases monotonically in the entire LET range and goes up to approximately 5–10 at very high LET^33^. For low LET instead merely DSB interaction on the µm scale causes most of the RBE increase. For increasing LET this process gets less important again due to saturation: If there are more than two DSB interacting within the sensitive volume the damage is not enhanced compared to when only two DSB interact within that domain, as in both cases a complex lesion on the µm scale is formed. Thus the relative effect per particle decreases and *RBE*_µm_ eventually drops below 1. The third modification by means of the *RBE*_10µm_ is a pure damping factor which can only take values smaller than 1. It gets relevant for high LET where individual tracks have a high cell inactivation capability so that cells are killed if they are hit at least once, or survive if they are not hit. Hence, the nuclear area distribution which determines the number of hits per nucleus becomes important. To gain further insight in the meaning of the effect decomposition for applications, we also considered the case when the RBE is plotted against the remaining range of the carbon ions in water up to where particles stop in the so-called Bragg peak as used in clinical applications such as heavy ion cancer therapy or in material sciences in the supplementary information (c.f. Fig. S1). As turns out, in the Bragg peak region, all scales are of importance and may not be neglected for a proper RBE prediction.

## Discussion

Concerning our experimental data, ignoring a µm lesion interaction mechanism precludes understanding the enormous effect enhancement we observed simply due to µm focusing of low LET protons (marked with ‘b’ in Fig. 2). In LEM, we attribute complex damage solely to the interaction of DSB on the µm scale. We also take the enhanced damage arising from the increased interaction of SSB to form DSB on the nanometer scale into account, but do not distinguish between varieties of nanometer DSB complexity, i.e additional single strand breaks in nanometer proximity to a DSB.

Considering the micrometer scale two distinct interpretations of the damage interaction become possible: Within the LEM the DSB interaction is regarded as a clustering of DSB within µm sized subcompartments of DNA strands, identified with chromatin loops of 2 Mbp size. These clustered DSB are supposed to be hard to repair correctly for the cell. Practically, cubic volumes were assumed as relevant domains, and their 510 nm size was obtained by compartmentizing the total nuclear volume in subvolumes corresponding to 2 Mbp DNA content. This size fits well with the assumed size of a single chromatin loop^34,35,16^.

In an alternative approach, the range of the DSB interaction can also be interpreted in terms of subdiffusional processes the ends of a DSB are subject to^36^, eventually leading to the formation of chromosomal aberrations. The subdiffusional processes lead to the high probability for the interaction of DNA ends being essential for a correct connection of the ends of a singular DSB and thus being relevant for DSB repair while normal diffusion would lead to a large number of unconnected DSB ends. However, this high probability to connect DNA ends gives also a high probability to connect the wrong ends if they are within the same distance as the ends being separated from a singular DSB. Thus, chromosomal aberrations are formed between the same and also between different chromosomes^24^ if placed side by side within the characteristic length of the subdiffusional process. The typical distance in which the ends will survey for other ends after induction is given by the root mean square σ of distances from the subdiffusion of DNA ends and DSB after a certain time span t^36^ as

3 $$\sigma =\sqrt{0.0033\,\mu {m}^{2}/{s}^{0.49}\cdot {t}^{0.49}}{\rm{.}}$$

A root mean square σ = 361 nm is obtained for the distances of two DSB that are randomly distributed within the assumed 510 nm cubic domains of LEM. This distance would be obtained by subdiffusion after a mean time of 1812 s according to Eq. 3, a reasonable time scale after which DSB ends may have found each other.

In any case, the distinction of isolated DSB and clustered DSB in the 510 nm domains proved to be extremely useful for describing effects for different endpoints and physical conditions such as orthovoltage or ultrasoft X-rays as well as temporal effects of radiation exposure^16,37,38^. With this the LEM stands in contrast to another important model approach formulated by Goodhead and Nikjoo, who highlight the importance of nm complexity in their model^14,39^: They introduce different varieties of nanometer DSB complexities such as a DSB accompanied by further SSB some bp apart while such nanometer DSB complexity is neglected in LEM. Their approach was successful in explaining the important role of secondary electrons. In particular when low energetic secondary electrons play a predominant role, considering micrometer size sensitive sites only was not sufficient to properly express radiation effects^17^. However, these ideas were not followed to consider multiple targets of different sizes, comprising both nm and µm scale.

The prominent role of the µm scale raises the question, to what relative extent the notion of nm and µm formed lesions contribute to overall damage, and in what aspects complexity plays a role on these scales. The data show that there are situations where apparently either the µm or nm scale plays a predominant role. The various features and limitations of different model approaches can help here to gain insight in what the most important processes are. Thereby it is remarkable that we succeed to find a reasonable model description of our data, despite the fact that our model has limits in neglecting different levels of complexity on the nm scale. Hence, our novel experimental findings and the interpretation in terms of different modelling approaches might represent a starting point to systematically analyze the emergence of complexity and the associated spatial scales.

The experimental data and the agreement of independent theoretic effect predictions of the LEM support the model hypothesis of three coexisting scales that determine cellular survival from DNA damage, and especially from radiation effects. Moreover, an analysis allows disentangling the relative contributions of the different mechanisms associated with the scales to the overall effect. Hence, considering the coexistent scales seems to be sufficient for an encompassing description of the full systematics of cell survival after DNA damage, as well as necessary for their prediction. Thus we propose that accounting for the three scales is a ‘must have criterion’ for a sound effect modelling, applicable to a broad variety of possible radiation qualities or other DNA damaging agents. We have to stress here that the LEM approach is designed to be the most simplistic, and does not claim to reflect the presumably rich diversity of complex lesions and details of the associated repair pathways^40^. However, it does claim to reveal the right proportion of complex to non-complex lesion formation for arbitrary ionization densities by probabilistic arguments. In this perspective there might be competing models with similar performance but a different mechanistic basis.

The findings of this study point to the main difference between the effects of DSB created in a correlated way (as e.g. by radiation) as compared to a more homogeneous distribution (as by other agents, e.g. hydrogen peroxide). As long as isolated DSB are formed by any kind of stress factor the relevance of such isolated DSB for affecting proliferation is small. Instead, the interaction of DSB and therefore their local concentration seems to be a prerequisite that DSB may lead to more severe consequences on the cell level.

In combination with previous findings, these observations suggest that different types of complex damage may exist, associated with different spatial extensions and biological targets, whose relevance depends on the distribution of more elementary DNA lesions.

Many projects in DNA repair research focus on revealing the nature of complex damage: Modern microscopy techniques exhibited clustering of damage markers such as γH2AX or their substructure, which might be associated to LET dependent damage complexity^41–43^. In addition, complementary to the spatial relevant scales addressed in the present work, various temporal scales are known to be of importance in DNA repair, and numerous approaches attempt to gain insight in their mechanistic underpinning. For example, it is well established that the time to repair DSB and the fraction of unrepaired DSB after one day of observation increases for high LET irradiation^44^, but can be modified by accumulating factors involved in DSB repair pathways^45,46^. Finally, at the level of connected tissues or organs, further spatial scales of radiation damage may occur, as is e.g. evident from recent experiments with so-called minibeams^47,48^, where very high doses of either low or high LET radiation are delivered in grids of tiny mm sized beams, while in between no irradiation is delivered. Such irradiation scenarios are usually well tolerated by various tissues, indicating intercellular repair processes on the mm scale and opening new perspectives for radiation therapy of cancer or other diseases.

Summarizing, all these approaches promise further insight in the role of complexity and repair of radiation damage. The present work formulates expectations for some of the properties of complex damage. The exploited microbeam techniques and theoretical effect modelling may play an important role in future studies.

## Methods

### Cell culture

The cell survival experiments were carried out using Chinese hamster ovary cells, CHO-K1. Standard cell culture conditions were used as described in the supplementary information. For cell survival experiments it is essential that all seeded cells get irradiated. For this purpose the cell cultivation setup was adapted and modified as described in^22^. A short description of the adaption is given in the supplementary information.

### Irradiation and dosimetry

Ion irradiation was performed at the Scanning Ion Microprobe SNAKE installed at the Munich 14 MV tandem accelerator^20^. The proton, lithium and carbon ion beams left the beam transport vacuum through 7.5 µm Kapton foil. After an air gap of ~30 µm and the cell carrier foil (5 µm) the ions hit the cells. The energy loss in the materials was calculated with TRIM, the LET values with SRIM^49^. Resulting energies and LET values at the target are given in Table 1.

The main parts of the irradiation setup and processes for spot irradiation have already been described in^21,22^. Spots of roughly 0.6 × 1.2 µm² were prepared by magnetic quadrupole focusing. The measured spot sizes for different beam times and the method of measurement are shown within Table 2. By electrostatically scanning fields of (500 × 500) µm² were irradiated with the given matrices. To ensure the requested number of particles at each spot a single/counted ion preparation method was used: During irradiation the ions were counted behind the sample and the beam was stopped by a fast electrostatic beam switch when the desired number of particles was reached. Then the next spot was addressed. To irradiate enough cells for the colony forming assay about 80–100 of these (500 × 500) µm² fields were stitched together to cover a circular area of ~4–5 mm diameter. An example of cells irradiated with a microbeam grid pattern is shown in Fig. S4.

To suppress systematic errors all broad beam irradiations were performed in the same setup. There the focused microbeam was scanned very fast many times over the (500 × 500) µm² field and particles were counted until the necessary number for the total area was reached before moving the cell sample to irradiate the next field. This approach allows switching immediately from spot to random irradiation under the otherwise same conditions. For all cell samples, the irradiation container was mounted in irradiation position, visualized by phase contrast microscopy and the size and position of the entire irradiation field were adjusted to cover the whole cultivation area. The total irradiation procedure takes 15–20 minutes.

The size of the irradiated spots was characterized for each experiment using fluorescent nuclear track detectors (FNTD) for protons and track etch detectors (polycarbonate or CR 39) for lithium and carbon ions, see Table 2. Details are given in the supplementary information.

### Survival assay

Immediately after irradiation the cells were removed from the mylar foil by trypsinization and cell concentration was determined using the Fuchs-Rosenthal cell counting chamber (NanoEntek). This chamber differs from other cell counting chambers by its large surface area and greater chamber depth. This design allowed accurate determination of small cell counts. Cell counting has been performed four times for each cell sample. The cell suspension was diluted in a dose-specific manner and reseeded into 12 well plates (Corning), where either 100 or 200 cells were seeded per well in typically 24 or 36 wells. The plates were incubated under standard culture conditions. On the fifth day after irradiation medium was removed from the wells and cells were rinsed once with PBS. The colonies were fixed with methanol and stained with 0.1% crystal violet. Plates were washed with water and then left for drying at room temperature. The counting of stained colonies has been performed by an automatic counting bioreader (Biosys). Only colonies consisting of 50 or more cells were counted as surviving cells. Colony numbers were mostly exceeding 10, assuring in combination with the large number of wells a sufficient statistical precision. The Plating Efficiency (PE) is described as the percentage of cells that are able to divide and form colonies. It was found to be 0.52 ± 0.08 from sham irradiated cells, where the uncertainty is the standard deviation among different beam times according to Table 2. The survival fraction of irradiated cells was calculated taking the PE of sham irradiated cells of the corresponding beam time into account.

### Data analysis

For all used ion species data from multiple irradiated samples obtained in multiple beam times are available (c.f. Table 2). First, from multiple irradiated samples within each beam time the average effect, i.e. average of the negative logarithms of the survival, was determined in order to calculate a representative survival value. Likewise uncertainties of the effects were obtained based on standard deviations of the measured effects: They are calculated as the square root of the sum of effect variances for irradiated and control samples, the latter of which have to be included as the control samples are used to calculate the plating efficiency. Second, thereby retrieved effects of different beam times were pooled and average values were calculated. Associated uncertainties were determined by Gaussian error propagation, i.e. as the square root of the uncertainty square sum. This pooling of data is justified as the spot sizes and all other conditions were comparable in the independent experiments carried out in the different beam times. Hence error bars in Fig. 2 and given uncertainty ranges in Table 1 are based on standard deviations and Gaussian error propagation, thus representing approximately 68% confidence intervals. For the x-ray reference curves the same procedure was applied. For the interpretation of the effects of spot irradiation the nuclear size distribution has been measured (Fig. S3) and the hit statistics, i.e. the frequency of hits per nucleus, were determined by Monte Carlo simulations. Further details on the data analysis are given in the supplementary information.

### Reference Radiation

Reference survival curves with X-rays have been determined using the same cell containers under identical culture conditions as for spot irradiations in order to have a proper characterization of the radiation sensitivity of the cells (see Fig. S2). The cells were exposed by a 200 kV X-ray irradiator irradiation facility (RS225, Gulmay Medical) with a dose rate of 1.21 Gy min^−1^ (15 mA) and a distance of 42 cm from the source to cells. The irradiation dose ranged from 0.5 to 8 Gy. Survival data of two independent experiments with at least triplicates are shown in Fig. S2. A least square fit reveals α = (0.156 ± 0.045) Gy^−1^ and β = (0.0235 ± 0.0055) Gy^−2^ and a covariance Cov(α, β) = 0.00025 Gy^−3^ for the common dose response curves obtained from both experiments. The uncertainties arise from the propagation of the fit parameter standard errors of the two independent experiments and can be interpreted as 68% confidence intervals.

### Local effect model predictions

The LEM predicts the effects of ion beam radiation based on (i) amorphous track structure, i.e. the local dose as a continuous function reflecting the local energy concentration averaged over a large ensemble of tracks., and (ii) the photon dose response curve for the endpoint under consideration. The latter is parameterized by the measured linear-quadratic coefficients α and β and the threshold dose D_t_, marking the transition to a linear dose response regime. According to an empiric relation^50^ the latter was chosen as D_t_ = 4 Gy + 1.1 α/β. Further details of the model steps are described in the supplementary information and in^26^.

For the present work the adapted LEM code for Gaussian beam spots allows a prediction of the effect of single spots to the cell nuclei. For this the geometric properties as determined from the nuclear size distribution were considered in the LEM calculations. The distribution of the number of spots to the nuclei has been obtained by a simple Monte Carlo algorithm as explained above. Using this information and the effect per spot the weighted overall survival was calculated.

LEM simulations have been performed individually for each beam time, taking into account the determined spot sizes. Uncertainties in the predicted effects were quantified from the uncertainties of the input photon parameters. In a next step the modelling results corresponding to individual beam times have been pooled in the same way as the experimental data, and uncertainties have been propagated. To substantiate our modelling results we verified via a sensitivity analysis by manually detuning model input parameters that within the uncertainty levels of the photon dose response parameters the general conclusions of our work did not change.

## Electronic supplementary material

Supplementary Information
