# Author Correction: DNA damage interactions on both nanometer and micrometer scale determine overall cellular damage

**DOI:** 10.1038/s41598-020-76568-3

**Published:** 2020-11-05

**Authors:** Thomas Friedrich, Katarina Ilicic, Christoph Greubel, Stefanie Girst, Judith Reindl, Matthias Sammer, Benjamin Schwarz, Christian Siebenwirth, Dietrich W. M. Walsh, Thomas E. Schmid, Michael Scholz, Günther Dollinger

**Affiliations:** 1 Department of Biophysics, GSI Helmholtz Center for Heavy Ion Research, Darmstadt, Germany; 2 TU München, Klinikum Rechts Der Isar, München, Germany; 3 Institute of Innovative Radiotherapy, Helmholtz Zentrum München, Neuherberg, Germany; 4 Universität Der Bundeswehr, München, Germany

Correction to:* Scientific Reports* https://doi.org/10.1038/s41598-018-34323-9, published online 30 October 2018

This Article contains an error in the legend of Table 2, where the axes width is incorrect.

“The beam spots had elliptical shape with semi-axes Δx and Δy, and different methods were performed for spot size characterization.”

should read:

“The beam spots had elliptical shape with axes Δx and Δy, and different methods were performed for spot size characterization.”

Additionally, there is an error in Figure 2, where the conversion between standard deviation and FWHM of a Gaussian distribution was performed by an incorrect factor. The correct Figure 2 appears below as Figure 1.

**Figure 1:**
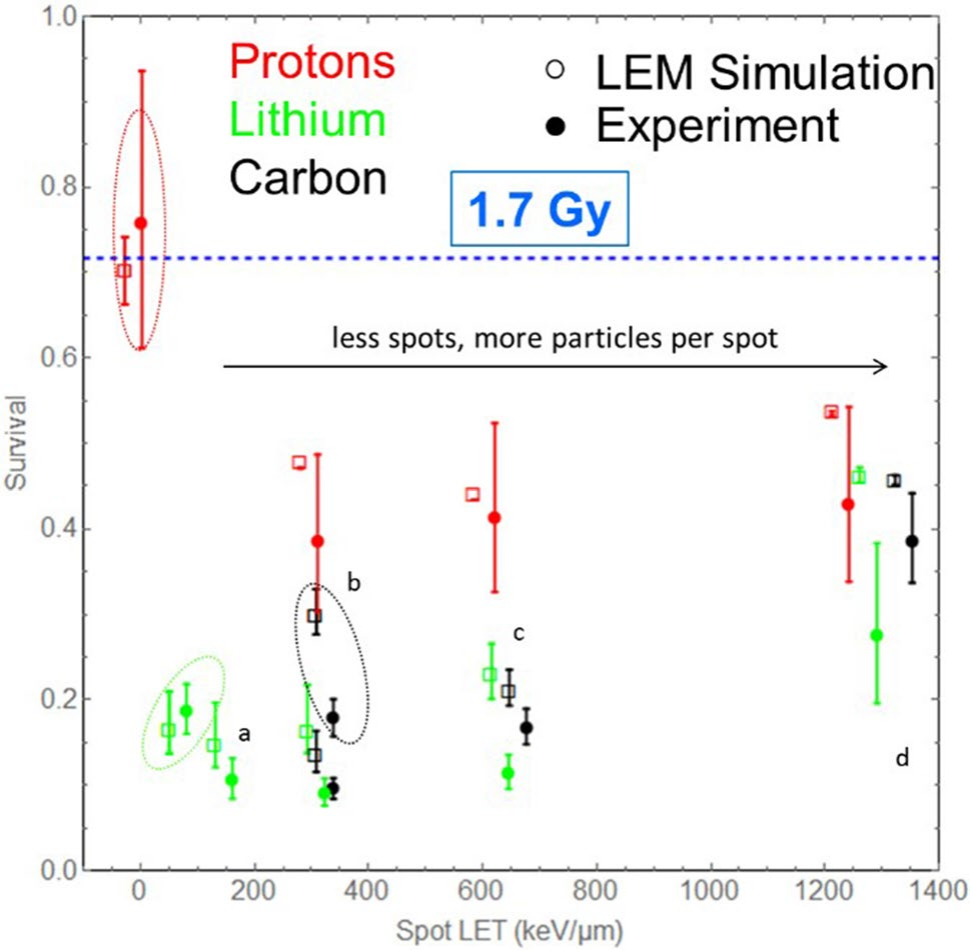
Cell survival vs the spot LET, i.e. the sum LET of all particles within a microbeam bunch for measurements (closed circles) and LEM simulations (open sqares) in comparison for different particles (p, Li and C in red, green and black, respectively) and different spot intensities. For all irradiations the dose was approximately 1.7 Gy (c.f. Table 1). Microbeam spots have been delivered as grids with mesh width of 3.82 µm (**a**), 5.4 µm (**b**), 7.64 µm (**c**) and 10.8 µm (**d**), where large mesh widths go along with larger particle numbers per spot. In addition, cell survival after broadbeam irradiation at 1.7 Gy is shown (data points are marked by dashed ellipses). The dashed blue line indicates the expected survival level after 1.7 Gy of X-rays. For better visibility simulation data points have been shifted by 30 keV/µm to the left. It is evident from the experiment that µm bunching enhances the effect, while at wider grids survival recovers again due to unhit cells. The simulations predict the survival in agreement with the measured data, supporting the underlying hypothesis. Note that the plot is shown in linear scale in order to present the differences at high survival most clearly.

