# Supplementary material for: DNA damage interactions on both nanometer and micrometer scale determine overall cellular damage
Source: Sci Rep. 2018 Oct 30;8:16063. doi: 10.1038/s41598-018-34323-9 (PMC6207695; doi:10.1038/s41598-018-34323-9)
Supplement: Supplementary file 1 — Supplementary Information [file 41598_2018_34323_MOESM1_ESM.docx]

**Supplementary Information**

**DNA damage interactions on both nanometer and micrometer scale determine overall cellular damage**

**Thomas Friedrich*^a^, Katarina Ilicic^b,c^, Christoph Greubel^d^**, **Stefanie Girst^d^, Judith Reindl^d^, Matthias Sammer^d^, Benjamin Schwarz^d^, Christian Siebenwirth^b,d^, Dietrich W. M. Walsh^d^, Thomas Ernst Schmid^b,c^, Michael Scholz^a^, and Günther Dollinger^d^**

^a^GSI Helmholtz Center for Heavy Ion Research, Department of Biophysics, Darmstadt,
Germany

^b^TU München, Klinikum Rechts der Isar, München, Germany

^c^Institute of Innovative Radiotherapy, Helmholtz Zentrum München,

Neuherberg, Germany

^d^Universität der Bundeswehr, München, Germany

*Corresponding author

Correspondence to: Thomas Friedrich
GSI Darmstadt
Planckstrasse 1
64291 Darmstadt
Mail: t.friedrich@gsi.de

Phone: +496159711340

**Results and discussion**

DSB statistics

As a primary step of the LEM effect calculations, the lesion statistics are evaluated. The results are shown in Tab. S1 for the different irradiation scenarios. The numbers demonstrate the manifestation of the basic model assumptions, where a distinction between isolated DSB (iDSB) and multiple, clustered DSB (cDSB) within chromatin loop domains are introduced. In particular, three features become apparent: First, simply focusing the low LET protons to spots in the 5.4 x 5.4 µm^2^ grid causes an almost six-fold increase in the number of cDSB. In the corresponding domains a cDSB typically comprises two DSB. While there are still many more iDSB than cDSB, the former are much less effective than the latter, explaining the tremendous breakdown in cell survival. Second, the overall number of DSB is higher for lithium ions as compared to protons, and even larger for carbon ions. The amplification factor of DSB induction due to the high local doses in the interior of the track structure, promoting SSB interaction on the nm scale, is about 2 for lithium ions and 4 for carbon ions. As a consequence the larger number of DSB also promotes the formation of cDSB. Finally, in the intense spots of the largest grid for each of the three particle species the number of cDSB is quite large. Hence hit cells are likely to be inactivated, while unhit cells survive, and the survival level is thus similar for all three particle species. The effect calculated from these numbers thus in general depends on the number and the proximity of DSB and the general presence or absence of tracks on cell nuclei.

Decomposing the effect into contributions from different scales

It is instructive to investigate the contributions of the different scales for carbon ions when the RBE in the low dose limit as shown in Fig. 3 is plotted against the remaining range of the carbon ions in water in Fig. S1, which comes close to a Bragg peak as often used in medical physics and material science applications (the difference is that here straggling and scattering corrections have been neglected). In the entrance channel where ions still have a large energy, the overall RBE is low and the most pronounced mechanism of effect enhancement is the DSB interaction on µm scale. In the Bragg peak region, shortly before the ions come to rest, also the SSB interaction has considerable importance, but still is somewhat less pronounced than the DSB interaction. There also the saturation correction (RBE_10 µm_) counteracts the effect enhancement. Practically this means that cells containing large nuclei will be inactivated more effectively than smaller ones.

Considering Fig. 2, the question arises why the µm scale appears to be so important for protons but not for lithium and carbon ions. For protons usually higher fluences are needed for the same effect, as the LET values are typically smaller compared to heavier ions at comparable depth. The larger number of hits per cell nucleus and lower local doses in the track centers imply that the µm scale predominantly is of relevance for protons and the other two scales are practically ignorable. This leads to an important conclusion: It demonstrates that the broad variation in effectiveness between radiation qualities goes back to the changing relevance of the spatial scales of DNA damage interaction.

It is worth to note here that in general RBE_nm_ is a factor which is purely derived from stochastics (33) and parameterizes initial damage, while RBE_µm_ additionally depends on repair capabilities of the cells, as it reflects in the low dose limit the RBE of individual particles after DNA damage processing. Hence, also the temporal scales associated with these factors are different. However, a further investigation of those is beyond the scope of this work.

**Methods**

Cell culture

For each experiment, an aliquot of frozen cells was thawed two weeks before the irradiation experiment. The cells were cultivated in RPMI-1640 medium (Sigma Aldrich) supplemented with 10% fetal calf serum (Sigma Aldrich), 100 units of Penicillin and 100µg of Streptomycin per ml culture medium, 2 mM L-Glutamine and 1 mM Sodium pyruvate (Sigma Aldrich). The cells were grown as monolayer culture at 37°C, 5 % CO_2_ and 95% humidity. The doubling time of CHO-K1 cells under these culture conditions was 12.2 ± 0.28 hours. On the day of the experiments, cells had been growing densely and were in a confluent state.

About four hours before irradiation cells were reseeded on mylar foil pre-treated with Cell-TAK (Corning) in an area at the center defined by an o-ring of 4 mm diameter. The total cell growth area was about 15 mm², which ensured a sufficient number of cells (about 10^4^ cells) after irradiation and reseeding in order to analyze clonogenic cell survival. Directly before irradiation the o-ring and its support are removed and cells are washed to remove all unattached cells.

The cell irradiation container consists of mylar foils clamped between three stainless steel plates with holes forming a closed volume. On one foil cells were grown. Thus, the ions passed through the cell sample and could be detected behind for single/counted ion preparation. The closed volume ensured a saturated atmosphere inside the container to prevent cells from drying out.

Determination of beam spot sizes at SNAKE

The size of the irradiated spots was characterized for each experiment. For 20 MeV protons fluorescent nuclear track detectors (FNTD) were used. Ionizing irradiation induced color centers in FNTD represent the beam spot size and shape and can be read out with confocal microscopy and evaluated as described in detail in (14). For the carbon and lithium beams, plastic nuclear track detectors are irradiated with a regular pattern and etched to make ion hits visible. The deviation of hit positions from the regular pattern reflects the beam spot size. Polycarbonate was used for carbon ions and CR39 for lithium ions. Measured spot sizes for various experiments are given in Tab. 1.

Reference radiation

Survival data after 200 kV x-ray irradiation of two independent experiments with at least triplicates is shown in Fig. S2. LEM uses the reference photon dose response parameters as given in the main text as input data to extrapolate the effectiveness of bunched or high LET particle radiation.

Hit statistics

For measuring nucleus size distribution CHO-K1 cells were seeded in the same cultivation setup parallel to irradiation experiments. After fixation with 2% paraformaldehyde (PFA) the nuclei were stained with DAPI after 15 minutes and z-stacks were acquired with confocal laser scanning microscopy. Projection along the z-axis was performed to get the largest shape of each nucleus in all z-slices. These shapes were outlined manually to determine the nucleus size (maximum area). Figure S3 shows the nuclear size distribution with a mean value of (67.6 ± 2.2) µm² and a standard deviation of the distribution of 24.8 µm². The size distribution was used for a Monte Carlo simulation of the hit statistics. There, individual shapes as determined above were placed randomly on the simulated irradiation matrix pattern for many times, and the number of hits inside the nuclei shapes was evaluated. The hit statistics considerations as well as the different beam application modes were verified with DSB markers as shown in Fig. S4.

Hypothesis underlying the Local Effect Model

In the LEM, in a first step the localized damage pattern after ion irradiation is derived from an amorphous track structure approach and a representative level of complexity (expressed as proportion of complex to non-complex lesions) is derived. The dose response of ion radiation is then calculated from the effect of a damage pattern of the same level of complexity induced by photon radiation. From the dose response corresponding linear quadratic parameters for ion irradiation are derived. The basic idea behind this concept is that locally the radiation action is mediated by means of secondary electrons, where only the local dose or local ionization density is of relevance for the induced lesions. Hence a mapping of photon effects to effects of ion radiation at the same local dose level is justified. Mathematical details of the model steps are described in (26).

We identify the amount of non-complex and complex lesions as the number of iDSB and cDSB as defined before, and propose a simple hypothesis on DNA damage induction and interaction which forms the basis of the model framework of the LEM and associated models. The simplicity of the underlying picture consists of only considering SSB and DSB as elementary lesions that interact on different scales to form more complex forms of damage and thus have much larger impact on the cell survival than the sum of the isolated damages would have. For low LET photon radiation SSB are assumed to be produced with a yield of 1250 SSB per Gy and cell nucleus while for DSB a yield of 30 DSB per Gy and cell nucleus is used. These values reflect typical experimental values, see (30) and references therein.

The hypothesized relevant spatial scales and associated mechanisms are illustrated in Fig. S5 and discussed in the following in more detail. On the first level, two SSB on opposite strands may form a DSB if they are induced sufficiently close to each other. This happens usually by correlated induction of SSB induced by the same secondary electron. For high ionization densities producing very high concentrations of secondary electrons as within high LET ion tracks, however, additional DSB are induced by a random coincidence if two SSB are induced by different secondary electrons in close neighborhood on opposite DNA strands. Thereby, SSB interaction is assumed to take place within 25 bp (corresponding to a linear distance on a DNA strand of 8.5 nm) and results in an enhanced DSB induction. The DSB yield for localized dose patterns increases with LET and can be derived from a simple stochastic model using these assumptions (33). While the DSB yield is almost constant up to about 100 Gy in line with experimental findings (32), LEM predicts that a local dose of about 8000 Gy results in a doubled DSB yield. The enhanced DSB yield implies a higher overall effect simply because more DSB are induced per Gy. The formation of additional DSB is visualized in Fig. S5 a.

On the next level the general idea is that isolated DSB induced within a chromatin loop of about 2 Mbp size can be repaired quite effectively, while multiple DSB within one loop, termed as clustered DSB, are more severe and will be repaired with less fidelity. Such lesions would disrupt the integrity of the DNA fiber and facilitate a formation of chromosome aberrations which might commit lethality when the cell enters into next mitosis. The typical size of chromatin loops would correspond to about half a micrometer, in line with the prior microdosimetric findings. Hence the DSB interact on the µm scale, leading to complex lesions associated with a high effectiveness. Again high ionization densities as within ion tracks promote the formation of such complex damage. Clustering of DSB in chromatin loops is visualized in Fig. S5 b.

Finally, for particle irradiation on the scale of the nuclear size, typically about 10 µm, the distribution of the number of particles that hit the nucleus matters. This can easily be verified by realizing that unhit cells will survive, as indicated in Fig. S5 c. So this mechanism is not driven by a pairwise lesion interaction process but rather by the frequency of missing lesions in a cell population, thus resulting in effect damping.

**Figures**


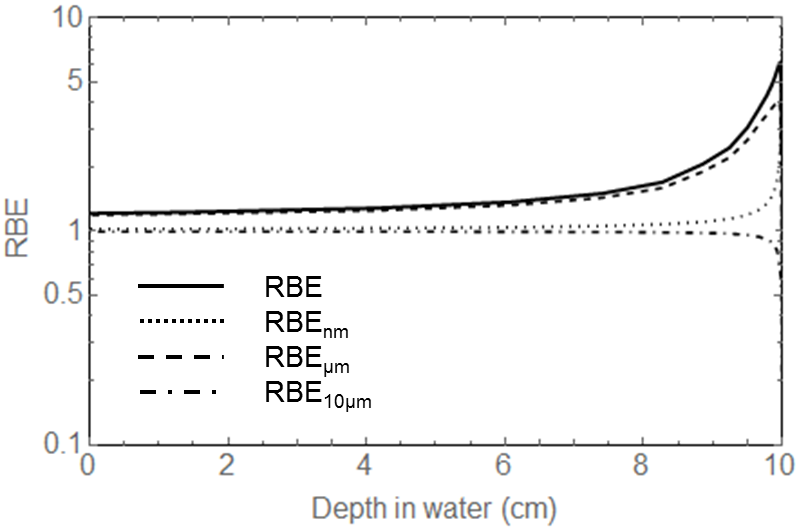


Fig. S1: Contributions of the different interaction mechanisms to the total RBE_α_ for monoenergetic carbon ions according to Eq. (2) plotted versus the depth in water. The curves have been calculated with the LEM for CHO cells as used in the described experiments. As in Fig. 3, in the high LET region close to the Bragg peak all three scales show a non-negligible contribution which has to be considered to understand the RBE.


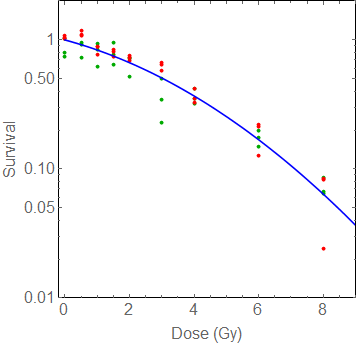


Fig. S2: Measured survival curves after 200 kV X-ray irradiation of two independent experiments (red and blue data points) and common fit curve according to linear-quadratic parameter entering the effect simulations.


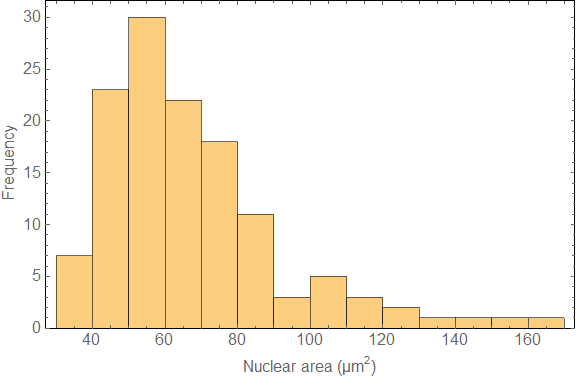


Fig. S3: Nuclear area distribution of the CHO cells used.


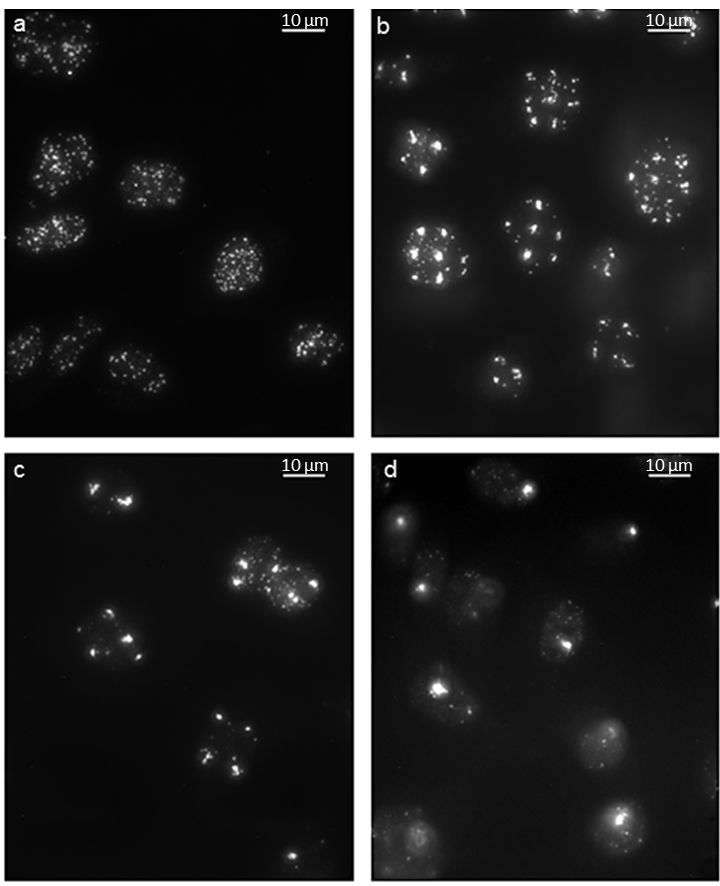


Fig. S4: Irradiation pattern in CHO-K1 cells visualized by γ-H2AX foci as a DSB marker 30 minutes post proton irradiation. A mean dose of 1.7 Gy was applied after a) random proton irradiation b) focusing 117 protons (20 MeV) to spots of a 5.4 x 5.4 µm² grid, c) focusing 234 protons to spots of a 7.6 x 7.6 µm² grid and d) focusing 468 protons to spots of a 10.8 x 10.8 µm² grid.


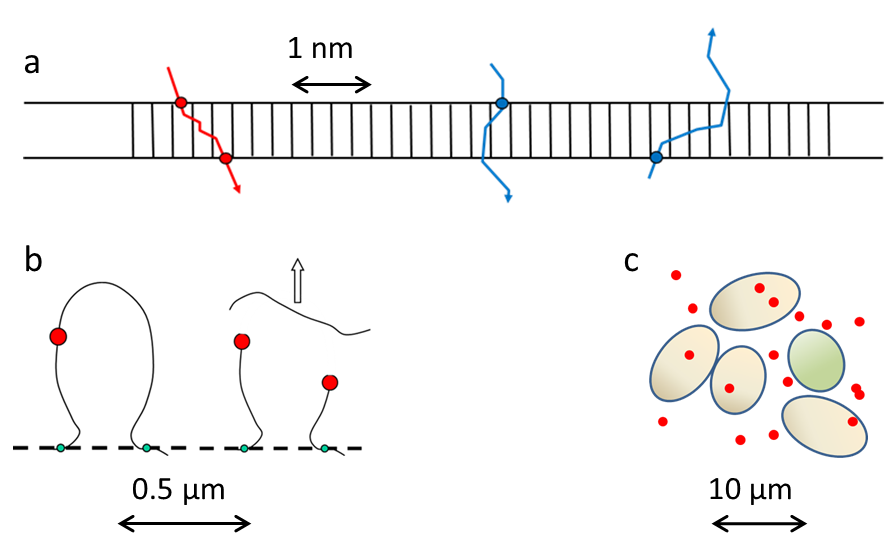


Fig. S5: Hypothesis of three coexisting relevant scales for radiation damage: On the nm scale (a) secondary electrons induce DSB composed of two SSB, either induced from one electron track or, at high local energy densities, by coincidence of different electron tracks. On the µm scale (b) damage is effective when two or more DSB are aggregated within DNA chromatin loops to form more complex damage, or if they are close enough to form chromosome aberrations. At the scale of the nuclei of about 10 µm (c) unhit cells survive while multiply hit nuclei accumulate damage.

**Tables**

Tab. S1: DSB statistics predicted by LEM for the various irradiation scenarios. The lesion numbers have been evaluated on the per particle, per spot or per cell basis, as average values over a large cell ensemble. The number of induced DSB per cell as well as the number of isolated DSB (iDSB, exactly one DSB in a 510 nm sized domain) and complex DSB (cDSB, more than one DSB in a 510 µm domain) per cell are given.

|  |  | #DSB  per particle | #DSB  per spot | #DSB per cell | #iDSB per cell | #cDSB  per cell |
| --- | --- | --- | --- | --- | --- | --- |
| X-ray | Random | - | - | 51 | 50.18 | 0.41 |
| p | Random | 0.19 | - | 51.34 | 50.51 | 0.42 |
| p | 5.4 x 5.4 µm^2^ | - | 22.23 | 50.55 | 45.13 | 2.61 |
| p | 7.64 x 7.64 µm^2^ | - | 44.46 | 50.32 | 40.99 | 4.39 |
| p | 10.8 x 10.8 µm^2^ | - | 88.92 | 50.60 | 34.27 | 7.12 |
| Li | Random | 11.03 | - | 97.85 | 49.85 | 20.78 |
| Li | 3.82 x 3.82 µm^2^ | - | 22.06 | 101.40 | 50.43 | 21.67 |
| Li | 5.4 x 5.4 µm^2^ | - | 44.12 | 103.76 | 45.09 | 24.50 |
| Li | 7.64 x 7.64 µm^2^ | - | 88.24 | 102.11 | 37.29 | 24.89 |
| Li | 10.8 x 10.8 µm^2^ | - | 176.48 | 101.73 | 26.86 | 26.05 |
| C | Random | 86.14 | - | 197.17 | 8.77 | 27.29 |
| C | 5.4 x 5.4 µm^2^ | - | 86.14 | 217.30 | 9.54 | 29.89 |
| C | 7.55 x 7.55 µm^2^ | - | 172.28 | 219.25 | 9.30 | 29.24 |
| C | 10.65 x 10.65 µm^2^ | - | 344.56 | 223.54 | 7.70 | 27.72 |
